# Assessment of Abdominal Constrictor’s Forces for Informing Computational Models of Orthostatic Hypotension

**DOI:** 10.3390/ma15093116

**Published:** 2022-04-26

**Authors:** Faiz Syed, Rejath Jose, Timothy Devine, Chris Coletti, Milan Toma

**Affiliations:** 1 Department of Osteopathic Manipulative Medicine, College of Osteopathic Medicine, New York Institute of Technology, Old Westbury, NY 11568, USA; fsyed09@nyit.edu (F.S.); rjose02@nyit.edu (R.J.); 2 Institute for Clinical Competence, College of Osteopathic Medicine, New York Institute of Technology, Old Westbury, NY 11568, USA; tdevine@nyit.edu (T.D.); ccoletti@nyit.edu (C.C.)

**Keywords:** orthostatic hypotension, abdominal constrictor, force loads, statistical analysis

## Abstract

Orthostatic hypotension is defined as a sudden drop in blood pressure upon standing from a sitting or supine position. The prevalence of this condition increases exponentially with age. Nonpharmacological treatments are always the first step in the management of this condition, such as the use of an abdominal constriction belt to optimize the blood volume in the abdomen. A multitude of clinical trials have shown the efficacy of elastic abdominal compression as well as compression using an inflatable bladder; however, there are currently few accessible consumer products that can provide abdominal compression by using an inflatable bladder that ensures the correct amount of pressure is being exerted on the subject. This study serves to quantitatively analyze forces exerted in inflatable abdominal binders, a novel treatment that fits the criterion for a first-line intervention for orthostatic hypotension. Quantitative values aim to indicate both the anatomic regions of the body subjected to the highest pressure by abdominal binding. Quantitative values will also create a model that can correlate the amount of compression on the subject with varying levels of pressure in the inflatable bladder. Inflatable binders of varying levels of inflation are used and localized pressure values are recorded at 5 different vertical points along the abdomen in the midsternal line and midclavicular line, at the locations of the splanchnic veins. These findings indicate both the differences in the compressive force applied through elastic and inflatable binding, as well the regions on the abdomen subject to the highest force load during compression by an abdominal binder. A medical manikin called the iStan Manikin was used to collect data. The pressure values on a manikin were sensed by the JUZO pressure monitor, a special device created for the purpose of measuring the force under compressive garments. The pressure inside the inflatable bladder was extrapolated from a pressure gauge and the pressure was recorded at different degrees of inflation of the belt (mmHG) along two different areas of the abdomen, the midsternal line and the midclavicular line, to discern differences in force exerted on the patient (mmHG). Computational studies on the data from the JUZO pressure monitor as well as the data from the pressure gauge on the inflatable bladder allow us to create a model that can correlate the amount of pressure in the inflatable bladder to the amount of pressure exerted on the belt, thus making sure that the patient is not being harmed by the compressive force. The results of our study indicate that there is no significant difference between the pressures exerted on the midsternal and midclavicular lines of the body by the abdominal binder and that no significant difference exists between the external pressure measured by the inflatable belt and the pressure sensed on the human body by the JUZO sensor; however, we were able to extrapolate an equation that can tell the user the amount of pressure that is actually being exerted on them based on the pressure in the inflatable bladder as recorded by the gauge.

## 1. Introduction

Orthostatic hypotension (OH) is a condition marked by a decrease in blood pressure when a person assumes a standing or head-up tilt position from a previous position of lying or sitting down [1,2,3]. The condition is characteristically diagnosed by a reduction of systolic blood pressure by 20 mmHG or diastolic blood pressure of 10 mmHG within 3 min of assuming a more upright position [4]. The prevalence of OH increases exponentially with age, particularly in those 75 years and older [5]. It is significantly positively associated with falls in older adults, underpinning the clinical relevance to test for an orthostatic blood pressure drop [6]. A review of OH was conducted in collaboration with the Department of Neurology at Harvard Medical School, King Abdulaziz University, Lotus Spine and Pain, and the Department of Neurology at Stanford Medical Center [2]. This study found that the prevalence of OH increases with age, and is cited as being relatively uncommon in people less than 65 years of age. It was also found that OH is exacerbated in warm environments which would induce vasodilation. The review analyzes thirteen studies and finds the all-cause mortality in a five-year follow-up period to be increased by 1.5 times in patients with OH. It also discusses treatment for OH and compressive garments were noted as an effective non-pharmacological intervention. Such a condition is often the result of the failure of the sympathetic vasoconstriction on blood vessels, resulting in decreased blood pressure. Symptoms of OH reflect cerebral hypoperfusion including light-headedness, dizziness, near-syncope, syncope, fatigue, leg-buckling, and cervical muscle ischemia [7]. Nonpharmacologic treatments, such as an abdominal constriction belt, are used first in the treatment of OH [8,9,10,11]. The nonpharmacological approach is aimed at optimizing blood volume, decreasing postural venous pooling, reducing heat and post-prandial induced vasodilation, emphasizing physical conditioning, and minimizing nocturnal diuresis [12]. Although nonpharmacological treatment is always the first step in the management of this condition, a considerable number of patients will require pharmacological therapies [13]. However, to date, there is little data to support a standardized or recommended treatment [14,15].

While a patient is lying or seated, venous blood pools in the abdomen [16]. Due to the effects of gravity on veins that are not able to constrict in patients with OH, a large volume of blood shifts downward when such a person assumes an erect or upright position after a period of sitting or lying down [17]. This reduces the venous return to the heart, and thus reduces stroke volume. One concept for a nonpharmacological treatment of OH is the use of abdominal compression in a patient to provide a mechanical force on the veins resulting in an increased stroke volume [12,18]. A clinical trial conducted by Dr. Adrianus Smit et al. at the University of Amsterdam tested the effectiveness of elastic abdominal compression on patients with OH. Their results indicated statistically significant results in that abdominal compression increased standing blood pressure through an increase in stroke volume [19]. Additionally, an abdominal binder that makes use of an inflatable binder has been designed for the treatment of OH [20]. This design has been tested in a clinical trial comparing its therapeutic effects to another testing group receiving midodrine, the standard pharmacological method of care in OH. This study concluded that abdominal binding was as effective as midodrine, a pharmacologic drug that physiologically constricts blood vessels to elevate blood pressure [21].

This study will build upon the current findings of abdominal compression being an effective treatment in OH by analyzing the forces exerted on a human abdomen through computational measures to ensure that the patient is not being harmed by the compressive force. Baudenbacher, et al. has already patented the technology for an adjustable belt with an inflatable bladder that can provide compression to the abdomen to prevent pooling of venous blood in order to treat orthostatic hypotension; however a safe consumer product of this same technology is not accessible to everyone. The current study will solve this problem by creating an affordable abdominal compression belt with an inflatable bladder. Preliminary studies conducted on manikins and ourselves indicated that the pressure in the inflatable bladder as measured by the attached pressure gauge does not correlate with the pressure exerted on the patient, and the pressure changes as the patient’s abdomen moves with breathing motion. This study aims to create a model that can provide the user with an accurate reading of the pressure that is being exerted on their abdomen as compared to the pressure in the inflatable bladder. The values reported here are to be used for subsequent studies of computational nature to further study, evaluate, and manage OH.

## 2. Materials and Methods

An inflatable abdominal binder was created by attaching the inflatable portion of a sphygmomanometer cuff to the inside of a velcro weightlifting belt from a company called Gabor fitness. The belt itself is 39 inches in length and the portion where the inflatable bladder of the sphygmomanometer is attached has a width of 6 inches. The sphygmomanometer is from a company called Santa Medical and has a pressure gauge and inflatable bulb attached. The inflatable portion of the sphygmomanometer is 5 ½ inches in width. The velcro on the sphygmomanometer attaches easily to the velcro of the weightlifting belt, thus allowing for easy assembly. The belt with the sphygmomanometer can be wrapped around the manikin and the inflatable bulb can inflate the bladder of the sphygmomanometer to a desired external pressure, and the attached pressure gauge can indicate how much pressure is in the bladder of the sphygmomanometer. The pressure exerted on the subject by the abdominal constriction belts will be measured by using the JUZO pressure monitor (Compression Innovations, Inc., Cuyahoga Falls, OH, USA), a tool with force sensors that was designed to objectively measure pressure under applied bandages and compression wraps.

**Figure 1:**
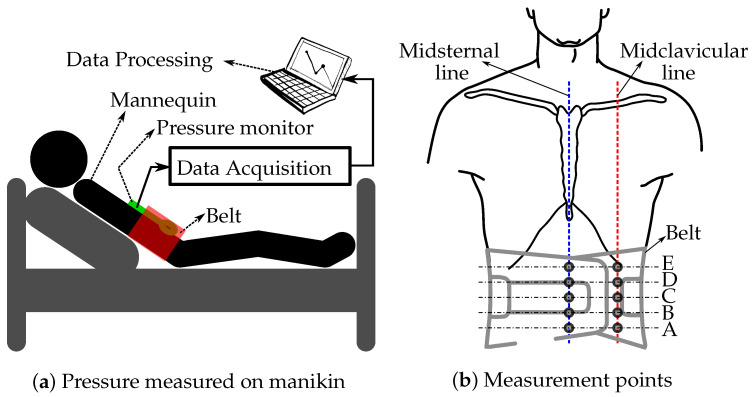
Illustration of the belt and pressure monitor placed on the manikin (**a**) and the locations where the pressure values under the inflated belt are measured along the midsternal and left midclavicular lines (**b**).

The subject of this study is a medical education manikin called CAE iStan manikin (serial number MMPH1010) (Canadian Aviation Electronics Ltd., Montreal, QC, Canada). The iStan is a wireless manikin that supports spontaneous breathing and asynchronous mechanical ventilation as well as a number of other features that can mimic the physiological changes in an actual patient. A medical manikin was used as a means to complete preliminary data collection to ensure the safety of the belt, before advancing to human subjects. The manikin will be supine with the upper body at a 45-degree angle. The belt with the sphygmomanometer will be fastened 2–3 inches below the Xiphoid process (bottom of sternum) of the subject, ensuring that the midpoint of the belt is aligned with the subject’s umbilicus (Figure 1a). In order to collect pressure data on different parts of the abdomen, the JUZO pressure monitor was first put on each of the points on the abdomen as illustrated in Figure 1b. There were 5 points, A–E, each along the midclavicular line and the midsternal line. Along the midsternal line, point C will be on the umbilicus and along the midclavicular line, point C will be a few inches lateral to the umbilicus.

Once the belt is fastened, the inflatable bulb of the sphygmomanometer was inflated to 20, 40, 60, and 80 mmHG and the pressure on the JUZO pressure monitor was measured. The procedure was repeated by repositioning the JUZO pressure monitor along points A–E along the midclavicular and midsternal lines.

## 3. Results

**Figure 2:**
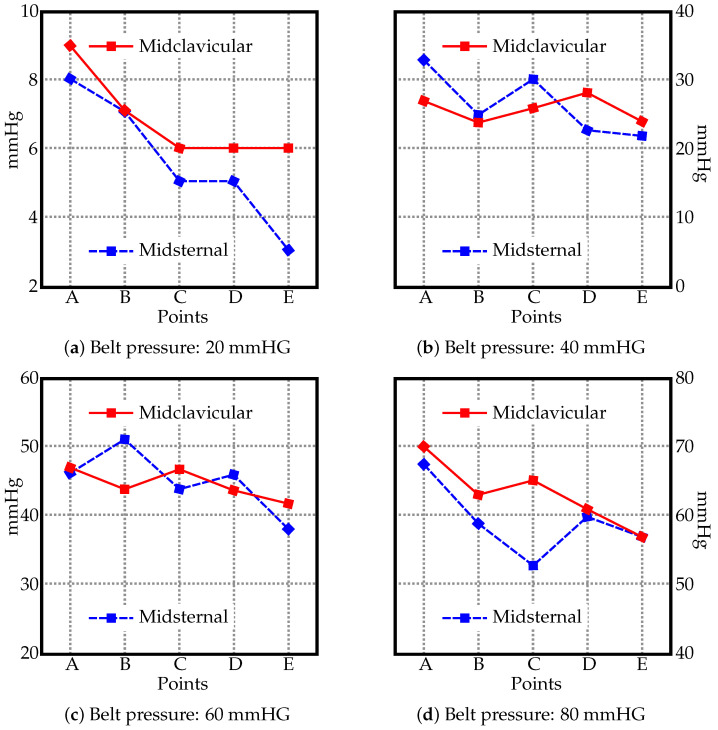
The pressure values [mmHG] along the midsternal and midclavicular lines at different points (see Figure 1) with the belt inflated to (**a**) 20 mmHG, (**b**) 40 mmHG, (**c**) 60 mmHG, and (**d**) 80 mmHG.

**Figure 3:**
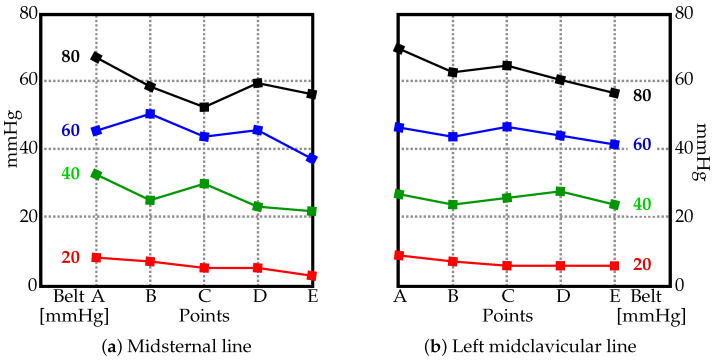
The pressure values [mmHG] along the (**a**) midsternal and (**b**) left midclavicular lines at different points (see Figure 1) with the belt inflated from 20 to 80 mmHG.

The pressure values measured using the JUZO pressure monitor and the pressure gauge of the sphygmomanometer are in Figure 2 and Figure 3. Figure 2 shows four charts (a–d), one per each value used to inflate the belt (20, 40, 60, and 80 mmHG). The two curves in each chart display the pressure measured along the midclavicular and misternal lines along points A–E. For comparison, the measured values from the JUZO pressure monitor at different belt pressures (20, 40, 60, and 80 mmHG) are reorganized in Figure 3 to clearly show the curves for the actual pressures exerted on the subject compared to the pressure recorded from the gauge on the belt. For sake of clarity, the data from the midclavicular line and midsternal line are separated into two. The data suggests a linear relationship between the pressure in the inflatable bladder and the actual pressure exerted on the subject. No significant trend can be observed in terms of differences in pressure exerted on the subject at different locations on the abdomen along the midclavicular and midsternal lines between points A–E.

**Figure 4:**
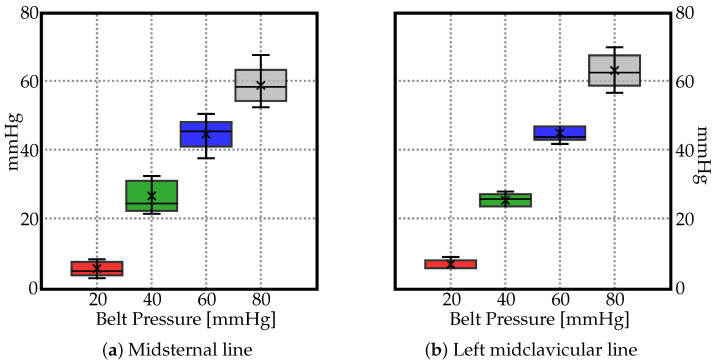
Box plots depicting groups of the data through their quartiles for (**a**) midsternal line, and (**b**) midcervical line. The ’×’ depicts the average values.

The measured pressure values are displayed in Figure 4 using box plot form depicting groups of the data through their quartiles. In the resulting charts, each rectangle was split into two parts, and thin T-shaped projections on each end. The bottom end is called the local minimum. Just above it is the bottom of the box, which marks the first quartile, that is, 25th percentile. The midline inside the box represents the median. The top of the box marks the 75th percentile. The distance between the top of the box and the top end (local maximum), barring any outliers, contains the final top 25% of the measured values. No outliers were identified in the data groups.

**Figure 5:**
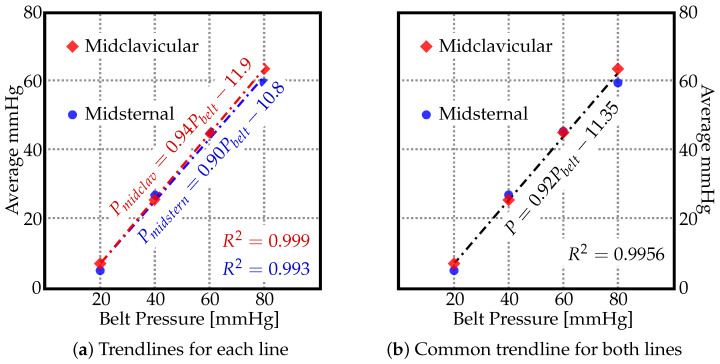
The average pressure values [mmHG] along the midsternal and midclavicular lines for different pressure values to which the belt was inflated ($P_{belt}$).

As no significant trend could be identified in Figure 2 and Figure 3, the average values (marked as ‘×’) in Figure 4 suggest a linear relationship between the pressure by which the belt is inflated and the pressure actually exerted on the human body along the midsternal and midcervical lines. To understand the exact relationship between them, the trendlines are depicted in Figure 5. The pressure as measured by the JUZO pressure monitor on points A–E were very similar for each chart in Figure 2, so an average value of points A–E was calculated for 20, 40, 60, and 80 mmHg. Figure 5 shows the average value as measured by the JUZO pressure monitor on the *Y* axis and the pressure in the sphygmomanometer (as measured by the pressure gauge) on the *X* axis. The blue points indicate the average values of points A–E for the midsternal line and the red points indicate values from the midclavicular line. Figure 5a shows the trendline separated for the dataset measured along the midclavicular and midsternal lines. However, because they appear to be very similar, a single trendline is calculated for all average values measured regardless of which of the two lines they belong to, as shown in Figure 5b. From the linear trendline in Figure 5b, the equation of the line of best fit can be extrapolated. The linear equation associated with the trendline in Figure 5b can be used to calculate the approximated average pressure values exerted on the subject as compared to the pressure in the inflatable bladder along the midsternal and midcervical lines.

Hence, the following empirical formula was found based on Figure 5b, where *P* is the pressure exerted on subject and $P_{belt}$ is the pressure in the inflatable bladder (either 20, 40, 60, or 80).

(1) $$P=0.92P_{belt}-11.35.$$

According to Equation (1), the approximated pressure values exerted on the human body along the midsternal and midclavicular lines can be calculated by multiplying the pressure inside the inflated belt by 0.92, and subtracting 11.35 from that value. The $R^{2}$ value for that linear equation is 0.9956, i.e., 99.56% of the variation in the output variable is explained by the input variables. Table 1 shows the comparison of average pressure values measured along the midsternal and midclavicular lines with the values calculated by using the empirical formula found from Figure 5b. The percent error is also indicated in Table 1, and the percent error for each of the points was below 5% except for the 20-mmHG point along the midsternal point. It is imperative to indicate that Equation (1) is valid only for the current model, and can be very different for different subjects. The current study is a step forward in creating a universal abdominal compression belt that can indicate how much pressure is being exerted on the user as compared to the amount the patient is inflating the belt.

## 4. Discussion

OH is often a side effect of a medication or a symptom of an illness. There are a number of clinical applications for a belt that will improve the lives of patients with OH. For example, patients with Parkinson’s disease often develop neurogenic OH due to cardiovascular sympathetic failure [22,23]. Presentation of neurogenic OH is very similar to normal OH with dizziness and syncope upon standing from a sitting position. Management of neurogenic OH is mostly supportive, focusing on preventing falls, increasing standing time and augmenting patients’ independence [22]. A low-cost, compressive belt would be an amazing addition to the management of patients with neurogenic OH. In a study by Dr. Alessandra Fanciulli, et al., the efficacy of elastic abdominal binders were assessed in Parkinson’s patients with neurogenic OH. There was no significant difference in supine mean blood pressure values when compared to placebo; however, symptoms of OH significantly improved during follow-up of these patients (*p* = 0.003), according to the OH Questionnaire [24]. Though there have been many advances in the management of OH, neurogenic OH remains a chronic, debilitating, and often progressively fatal condition [25].

Pharmacological OH is also very common. Many anti-hypertensive drugs often can cause first-dose OH, and combination of antihypertensives can cause OH. There are a number of pharmacological agents that can help patients with drug-related OH; however the evidence on the efficacy of many of these drugs are limited and cannot be used for hypertensive patients [26]. An abdominal belt might be a realistic alternative for patients taking antihypertensives suffering from OH due to its localized effect that can be attenuated anytime by removing the belt. As mentioned before, an abdominal belt was just as effective as midodrine for treatment of OH [21]. This could also improve patient outcome as the patient will not be suffering from polypharmacy and the side effects of multiple drugs. Management of OH is aimed at improving quality of life and reducing symptoms rather than at normalizing blood pressure. Nonpharmacologic measures are the key to success [27].

The current study correlates the relationship between pressure exerted on a subject and the pressure in the inflatable bladder. We extrapolated Equation (1), which indicates how different pressures, as measured by the sphygmomanometer, can correlate with actual pressure exerted on the patient. Equation (1) was compared to the actual values and that is indicated in Table 1. The percent error for each of the points was below 5% except for the 20-mmHg point along the midsternal point, thus indicating the accuracy of the model. However, Equation (1) is only valid for the current model, using the same materials as used in the current study. Further evaluation is needed on how Equation (1) would change for different subjects with different body proportions. Real-life application of the current study can be in the form of creation of a universal abdominal compression belt that is connected to an arduino or raspberry pi that will automatically create a mathematical model that can predict the amount of compression exerted on the user’s body as the user pumps the inflatable bladder. The current study is one step forward to creating such a universal belt. The next step would be to continue the experiment on patients and see the efficacy of the belt on actually raising blood pressure as well as OH symptoms. A number of studies have proven that abdominal compression is effective in improving OH if the compressive force is exerted right before rising, but further compression upon standing has no benefit [28]; however, there are limited studies on abdominal compression using an inflatable cuff as well the efficacy of applying compression when the body is in different positions. Randomized data evaluating the impact of therapeutic interventions on morbidity and mortality outcomes are lacking and it remains unclear whether OH treatment could improve prognoses [29].

Manikin simulators are used in healthcare to provide realistic patient environments for providers to practice skills and procedures while mimicking the human physiology response. This is known as fidelity. A high-fidelity manikin will provide the learner with the ability to go wrong without the risk of harm to a patient while providing lifelike responses. The lifelike features and responsive physiology allow learners and researchers to develop critical thinking, communication, clinical skills, and conduct research. They also provide the ability to reproduce high-acuity, low-volume patients, improvement of their psycho motor skills, and aides in confidence of the individual learner or team. Findings from manikin-based studies are commonly directly extrapolated to clinical practice [30]. Hence, for the purpose of this study, to maintain its simplicity and to keep as many indirect parameters constant as possible, the use of a medical manikin that was designed to simulate real patients to train physicians was chosen over an in-vivo or in-vitro study. Using this manikin creates a model that can accurately compute the pressure being exerted by the abdominal constriction belt without running the risk of harming a real person in the trial with the belt; however, after creating a working model that can automatically compare exerted pressures and that can ensure that the subject will not be harmed, testing on real subjects can start.

There are some limitations that should be considered with regard to this study. One of the limitations to the study was financial. The major limitation of the study would be testing on manikins instead of real life subjects. Even though the manikin provides a good proxy to assess the safety of the current model, a study on real-life subjects is warranted to further validate the study. This study was also limited by sample size as all of the results were collected on the same medical manikin with the same breathing patterns of 15 breaths per minute.

## 5. Conclusions

The purpose of subsequent studies is to develop, validate, and explore the use of computational algorithms to advance our understanding of OH and its management. This study advances existing knowledge, patents, and studies demonstrating the effects of abdominal constriction on OH, as now we are able to justify the areas of the abdomen subjected to the greatest force load and compare the internal pressure exerted on the abdomen by the constriction of the belt. Our results indicated that there was no significant difference between the force exerted on the different points of the abdomen along the midsternal and midclavicular lines; however, there was a linear relationship between the pressure exerted on the patient and the pressure in the inflatable bladder along the midclavicular line and midsternal line. This linear relationship was graphed and the line of best fit was extrapolated and Equation (1) was created, which can predict the amount of pressure exerted on the abdomen as compared to the pressure inside the inflatable bladder. This result can help guide the creation of a universal abdominal constriction belt designed for OH that can work on any user. The clinical use of the abdominal constriction belt in OH will help alleviate symptoms of OH when a patient assumes a more upright position after long periods of sitting or lying down. With the results of the study, belts can be better designed to exert more suitable pressures based on abdominal locations. Further studies can use the computational model demonstrated in this study and create a system that can automatically calculate the amount of pressure exerted on the subject. Further studies on real-life patients and under different conditions and variables can help us understand more about the use of non-pharmacological treatments for OH.

## Figures and Tables

**Table 1:** The average pressure values [mmHG] measured along the midsternal and midclavicular lines compared with the empirical formula found.

		Midsternal Line		Midclavicular Line	
Belt Pressure $P_{\mathrm{belt}}$ (mmHG)	Calculated $0.92P_{\mathrm{belt}}-11.35$	Measured (mmHG)	Error %	Measured (mmHG)	Error %
20	7.05	5.6	20.57	6.8	3.55
40	25.45	26.6	4.52	25.8	1.38
60	43.85	45	2.62	44.8	2.17
80	62.25	59.4	4.58	63.2	1.53

## Data Availability

Not applicable.

## Abbreviations

The following abbreviations are used in this manuscript:

OH	Orthostatic Hypotension
